# Exploring the whole standard operating procedure for phage therapy in clinical practice

**DOI:** 10.1186/s12967-019-2120-z

**Published:** 2019-11-14

**Authors:** Zelin Cui, Xiaokui Guo, Tingting Feng, Li Li

**Affiliations:** 1 Department of Laboratory Medicine, Shanghai General Hospital, 6th Floor, No. 3 Building, 100# Haining Road, Shanghai, 200080 China; 2 Department of Microbiology, Perelman School of Medicine, University of Pennsylvania, Pennsylvania, PA 19104 USA; 3 Department of Immunology and Microbiology, Shanghai Jiao Tong University School of Medicine, Shanghai, 200025 China; 4 Department of Clinical Pharmacy, Shanghai General Hospital, Shanghai, 200080 China

**Keywords:** Phage therapy, Clinical practice, Streamline, Standard operating procedures, Antibiotic resistance

## Abstract

We have entered the post-antibiotic era. Phage therapy has recently been given renewed attention because bacteriophages are easily available and can kill bacteria. Many reports have demonstrated successful phage treatment of bacterial infection, whereas some studies have shown that phage therapy is not as effective as expected. In general, establishment of a standard operating procedure will ensure the success of phage therapy. In this paper, the whole operating procedure for phage therapy in clinical practice is explored and analyzed to comprehensively understand the success of using phage for the treatment of bacterial infectious disease in the future. The procedure includes the following: enrollment of patients for phage therapy; establishment of phage libraries; pathogenic bacterial isolation and identification; screening for effective phages against pathogenic bacteria; phage formulation preparation; phage preparation administration strategy and route; monitoring the efficacy of phage therapy; and detection of the emergence of phage-resistant strains. Finally, we outline the whole standard operating procedure for phage therapy in clinical practice. It is believed that phage therapy will be used successfully, especially in personalized medicine for the treatment of bacterial infectious diseases. Hopefully, this procedure will provide support for the entry of phage therapy into the clinic as soon as possible.

## Introduction

Humans have entered the “post-antibiotic” era, in which existing antibacterial agents fail to kill drug-resistant bacteria that cause infections [1, 2]. Due to the wide use of antibiotics as additives in animal husbandry, the abuse of broad-spectrum antibiotics in clinical practice [3], and the convenience of travel among countries, the evolution and dissemination of antibiotic-resistant bacteria have accelerated [2]. As antibiotic resistance in bacteria becomes serious, scientists are exploring new strategies to control bacterial infection, especially infections caused by pandrug-resistant bacteria [1, 4, 5]. Antibiotic-resistant bacteria have evolved from initial multidrug-resistant bacteria to pandrug-resistant bacteria; multidrug-resistant bacteria are defined as having acquired non-susceptibility to at least one agent in three or more antibacterial categories, whereas pandrug-resistant bacteria are defined as not being susceptible to any agent in all antibacterial categories [6]. The effects of this situation include the death of patients and great economic burdens every year [7, 8].

Phages can kill bacteria and have been used for treating infections, including diarrhea and skin infections caused by *Shigella dysenteriae* and *Staphylococcus aureus*, respectively [9]. However, the discovery of antibiotics, subsequent large-scale industrial production, and wide clinical application have caused phage therapy to become unfavorable [4, 10]. Furthermore, studies of phage therapy nearly stopped for a long time in part due to its largely controversial efficacy [11].

Nonetheless, phage therapy has been extensively explored for the treatment of bacterial infections in former Soviet Union countries [12, 13]. However, as the Soviet Union collapsed, the substantial achievements of phage therapy research in these countries were not well recognized in the current English-dominated global scientific publication society [13]. The reason is partially because most of these studies were not performed according to Western standards (e.g., nonrandomized and non-placebo controlled) [14]. In addition, there were concerns related to endotoxin contamination and inconsistency of the results of phage therapy trials [12]. Moreover, phage therapy was scientifically unattractive in the USA due to political circumstances [15].

In recent years, phage therapy has been re-emphasized as the severity of drug-resistant bacterial infections has increased [16]. Indeed, phages alone or combined with antibiotics have been successfully used to treat different bacterial infections [1, 17, 18], including bloodstream infection [1], lung infection, chronic otitis, skin burn infection [19], and enteric infection [17]. In contrast, other clinical reports have shown that phages are not as effective as anticipated, both for topical bacterial infection and ETEC (Enterotoxigenic *Escherichia coli*)-associated diarrhea [20, 21], possibly due to insufficient phage titers or coverage. Furthermore, published reports indicate no side effects observed in clinical trials [22, 23]. A current study describes the production of a phage cocktail for use in clinical trials [22], and phage preparations are already entering into clinical trials [20].

It is well known that phages are different from traditional chemical antibacterial drugs. Traditional antibacterial drugs are usually used for treatment based on a physician’s experiences and/or according to the bacterium’s antibiotic resistance profile [24]. Phages have their own specificities; they consist of protein components, and the genome that encodes genomic information, their bactericidal activities and replication requires hijacking of the metabolism of host bacterial cells [25]. Some phage-derived proteins are able to increase platelet activation and are associated with mortality in patients with invasive pneumococcal disease [26]. Here, the entire procedure of phage therapy in clinical practice is outlined and analyzed to support the entry of phage therapy into the clinic as soon as possible.

### Isolation and identification of bacteria causing infection

The isolation and identification of pathogenic bacteria causing infection are prerequisites for the success of phage therapy. In clinical practice, once a patient is suspected to have a bacterial infection, the bacteria must be isolated and identified. At the same time, nonbacterial infection cases (such as viral, fungal or parasite infections) should be excluded from phage therapy. With the development of diagnostic testing techniques, many new nonculture-based methods are being applied in clinical laboratories for diagnosis of bacterial infection, including MALDI-TOP MS, PCR, gene sequencing, and immunological methods [27]. For patients who choose phage therapy, the first step is to isolate the pathogenic bacterium using culture-based methods. Next, the pathogen can be identified by nonculture-based methods. Finally, the sensitivity of the isolate to phages is screened for therapy. Phage therapy may be considered based on the information obtained in the above steps. In other words, phage therapy should not be recommended to treat a bacterial infection that was directly diagnosed by nonculture-based methods.

Additionally, a phage approach may be chosen when traditional antibacterial agents are useless for therapy. It is well known that antibiotic preparations, administration routes, dosages, and frequencies have been well established for different types of bacterial infections, as have metabolism and side effects, among others. Indeed, these factors have been practically clarified for humans. The primary choice for the treatment of a bacterial infection is to use the standard antibacterial drug treatment based on the bacterial drug-resistance profile and/or a physician’s experiences [24]. Therefore, a phage would be advised only when antibacterial drugs are proven to be ineffective and/or when the infection is caused by multidrug-resistant or pandrug-resistant bacteria. Currently, traditional antibacterial drugs remain the first choice for treating a bacterial infection; phage can be viewed as a supplement to traditional drugs, as it is difficult to completely replace antibiotics.

### Establishment of a phage library and determination of phage-sensitive bacteria

Establishment of a library containing a variety of phages is the foundation of success for antibacterial therapy. Bacteriophage specificity is strict, and their host range varies from extremely narrow to broad [28–30]. The library should contain different lytic phages that are able to kill different species and a variety of virulent phages that can kill the same strain [31]. Most importantly, it is necessary to set strict enrollment criteria for phages in clinical use. To avoid potential risks, only phages that meet the criteria should be assessed. The guiding principles of safe phage therapy are ① safety at the genomic level, with ‘no undesired genes, such as toxin genes, antibiotic-resistance genes etc.’, and ② use of lytic phages [32, 33]. The library should include phages targeting epidemic multidrug-resistant bacteria species, including *Acinetobacter baumannii*, *Klebsiella pneumoniae*, *Pseudomonas aeruginosa*, *Escherichia coli*, *Staphylococcus aureus*, and *Enterococcus* [34, 35].

Phage-sensitive bacterial confirmation is the precondition for the success of antibacterial therapy. First, the bacterium causing the infection must be obtained and identified; second, phages in the library that are effective against the pathogenic bacterium must be screened and confirmed. This process indicates that a standard method to test the phage sensitivity of bacteria should be established. To date, the most favorable standard method is the two-layer agar plate because it is a simple and straightforward method [28]. If there are different phages in the library that kill the same bacterial strain, cocktails containing these phages are preferred. Reports have shown that phage cocktail preparations might enhance bactericidal efficacy and also reduce the probability of the emergence of phage-resistant isolates during therapy [1, 36].

### Preparation, storage, and transport of phage preparations

The development of standard operating procedures for phage preparations as well as storage and transport are required for phage therapy in clinical practice. Currently, there are no reported standard operating procedures for phage preparation in clinical use. The available reported phage therapy clinical trials included several necessary steps for their application: phage isolation, phage characterization, phage susceptibility testing, endotoxin removal, and relevant product production [22]. A reported case of successful phage therapy also further included bacteriophage-resistant bacteria monitoring and evaluation of bacteriophage pharmacokinetics during therapy [1]. However, a recent double-blind phase 1/2 trial did not include bacteriophage-resistant bacteria monitoring and bacteriophage pharmacokinetics or endotoxin removal; in fact, worse phage agents were diluted to lower titers to meet the clinical limits of endotoxin [20]. Our goal is to systematically suggest a standard procedure for phage application in the clinic. First, a suitable sensitive host bacterium in which the phage may efficiently synthesize components for amplification must be selected. One approach is to choose bacteria that cause infection and thus can artificially select phage clones that more effectively kill their host bacteria, as phage microevolution during amplification and artificial selection of more obvious plaques (phage clones) may ultimately enhance their bactericidal activity [37]. In addition, to reduce the possibility of packaging undesired bacterial genomic fragments containing genes into phage particles during amplification [38], the host bacterium must be free of virulence genes and antibiotic-resistance genes, thus avoiding the possibility of disseminating these genes by the phage when used clinically.

Second, to facilitate direct contact of the phage with the pathogenic bacterium, different types of phage preparations (e.g., injection, powder, cream) should be developed for special bacterial infections [39]. For example, aerosolized phage preparations may be chosen for the treatment of respiratory tract infections [40–42]. The preliminary purification steps for obtaining purified intact phage particles include bacterial lysis and phage particle release from the host cell, phage precipitation, discontinuous density gradient centrifugation, continuous density gradient centrifugation, and/or chromatography technology [43]. Phage preparations should be endotoxin free, kept intact with high titers [44], and meet criteria for clinical use [45, 46]. Proper storage and transport are also required. Phage preparations are similar to other biological products, and extreme environmental conditions should be avoided; phages need to be protected from high temperature and extreme acidic or alkaline conditions [28]. For example, phages can be stored at pH 7.35–7.45 at low temperature and should not be placed at temperatures higher than 37 °C. The phage stock also should not be refrozen and rethawed [47].

### Administration strategy of phage preparation

The outcomes of phage therapy are particularly dependent on the administration strategy. Similar to antibiotic therapy, the approach can accelerate the evolution of antibiotic-resistant strains [48, 49]. Phage-resistant bacteria can also evolve and emerge during therapy, which would lead to treatment failure [50]. To decelerate the evolution and emergence of phage-resistant strains, cocktails containing different phages that are effective against sensitive bacteria are a priority for therapy [1, 18]. This method decreases the probability that bacteria simultaneously evolve resistance to different phages [36].

Regardless, phage-resistant clones do occur. At the same time, their antibiotic-resistance profile may change [51]. Thus, the combination of phage and antibiotic administration is a strategy for combating these bacteria [52]. In general, co-administration of phages and antibiotics should primarily consider the presence of an asynergistic bactericidal effect. In addition, a high-frequency and -dose administration strategy is preferred because a low dose of phage with a low frequency may not efficiently and completely kill the bacterium [20]; low dosages may also accelerate the evolution and emergence of phage-resistant isolates [53]. Thus, a cocktail with a high-dose and high-frequency administration strategy is recommended for optimal therapeutic effects.

### Administration route of phage preparations

The best administration route for phage preparations may facilitate sufficient phages coming into direct contact with bacteria. In addition, any other treatment operations that would inactivate the phage should be avoided during therapy. For example, in the case of gastrointestinal bacterial infection, inactivation by stomach acid should be avoided when the phage is given orally [54]. Other treatment measurements that may potentially inactivate phages, such as antiseptic agents, should also not be used with phage preparations [55]. Furthermore, phages are nanoparticle sized, suggesting that they may not have effective diffusion efficiency in the human body, though the diffusion efficiency of phages in human tissues is still unclear. Therefore, the best administration route of a phage preparation is one in which the particles are delivered in a manner to directly contact the pathogenic bacteria. As an example, a phage powder, phage-containing lotion or dry gauze layer containing phages could be considered for skin infections (Table 1) [17]; aerosolized phage preparations may be chosen for respiratory tract infections [40–42]; phage infusion preparations may be primarily considered for bloodstream infection [1]; and capsules containing phages that can protect the particles from inactivation by stomach acid should be preferred for gastrointestinal infections [54], though the effect of the capsule protecting the phage from inactivation in the stomach should be evaluated first [54]. Regardless of the formulation for phage preparations and the types of bacterial infections they are used to treat, the ideal administration route and/or phage preparation will ensure that sufficient intact phages successfully gain access to sites of infection and directly contact the pathogenic bacteria.

**Table 1:** Administration routes of different phage preparations for various bacterial infections

Infections	Phage preparations	Administration routes
Blood	Infusion preparations	Intravenous
Enteric	Capsule	Oral
Skin	Powder, lotion or dry gauze layer	Spray, washing, coating
Vaginal tract	Tablets	Imbedding
Respiratory tract	Aerosolized preparations	Inhalation

### Monitoring efficacy of phage therapy and detecting emergence of phage-resistant strains

The emergence of phage-resistant strains should be monitored regularly during phage therapy. The patient’s conditions also need to be observed regularly to evaluate whether they are improving, and clinical samples from bacterial infection sites should be assessed in a timely manner to evaluate therapeutic efficacy, the emergence of phage-resistant strains and efficient phage titers. Phages should be replaced once particular phage-resistant bacteria emerge. If there are no phages in the library that kill a phage-resistant bacterial strain, the strain can be further used as a host bacterium to screen various types of samples (e.g., soils, feces) to isolate new effective phages. Such new phages can be added to continuously enrich the library if they meet the criteria, and the may be prepared using the method mentioned above. In theory, phage therapy can be considered as an example of personalized medicine for bacterial infections [1]. In addition, reports have shown that phage-resistant strains sometimes become less virulent [56, 57] and/or are associated with changes in surface-related components of bacteria [58]. Phage resistance may also be accompanied by changes in antibiotic resistance [51]. Therefore, the antibiotic resistance profile of phage-resistant strains should be simultaneously tested. The synergistic bactericidal activity of combining phages and antibiotics in the clinic should also be studied [59], and further treatment strategies using phages alone and/or in combination with antibacterial drugs should be considered based on the results.

## Conclusion and perspectives

In this paper, we summarized and analyzed the whole procedures of phage therapy, and the key points of these procedures were analyzed. The establishment of a phage library is the foundation of a successful therapy. Because of phage specificity, one type of commercial phage preparation cannot be effective for different bacterial infections, even for infection with the same strain. Moreover, epidemic bacteria differ by region. It is well known that phages have strict specificity for bacteria, similar to the Chinese philosophy of “Yin” and “Yang”. On the one hand, the specificity of a phage limits its use for therapy. However, the shortcomings can be overcome by establishing a phage library and preparing cocktails to broaden the bactericidal range. On the other hand, with the emphasis of personalized and translational medicine, phage specificities also highlight their advantages, and they can be seen as personalized medicine for specific bacterial infections. For example, phages do not disturb commensal bacterial composition [60]. The success of treating a bloodstream infection caused by pandrug-resistant *A. baumannii* with phage in 2017 was based on a phage library established in advance [1]. Ultimately, we call for all of phage researchers around the world to share phages and to establish phage libraries in different regions to promote the success of phage therapy in the future.

**Fig. 1:**
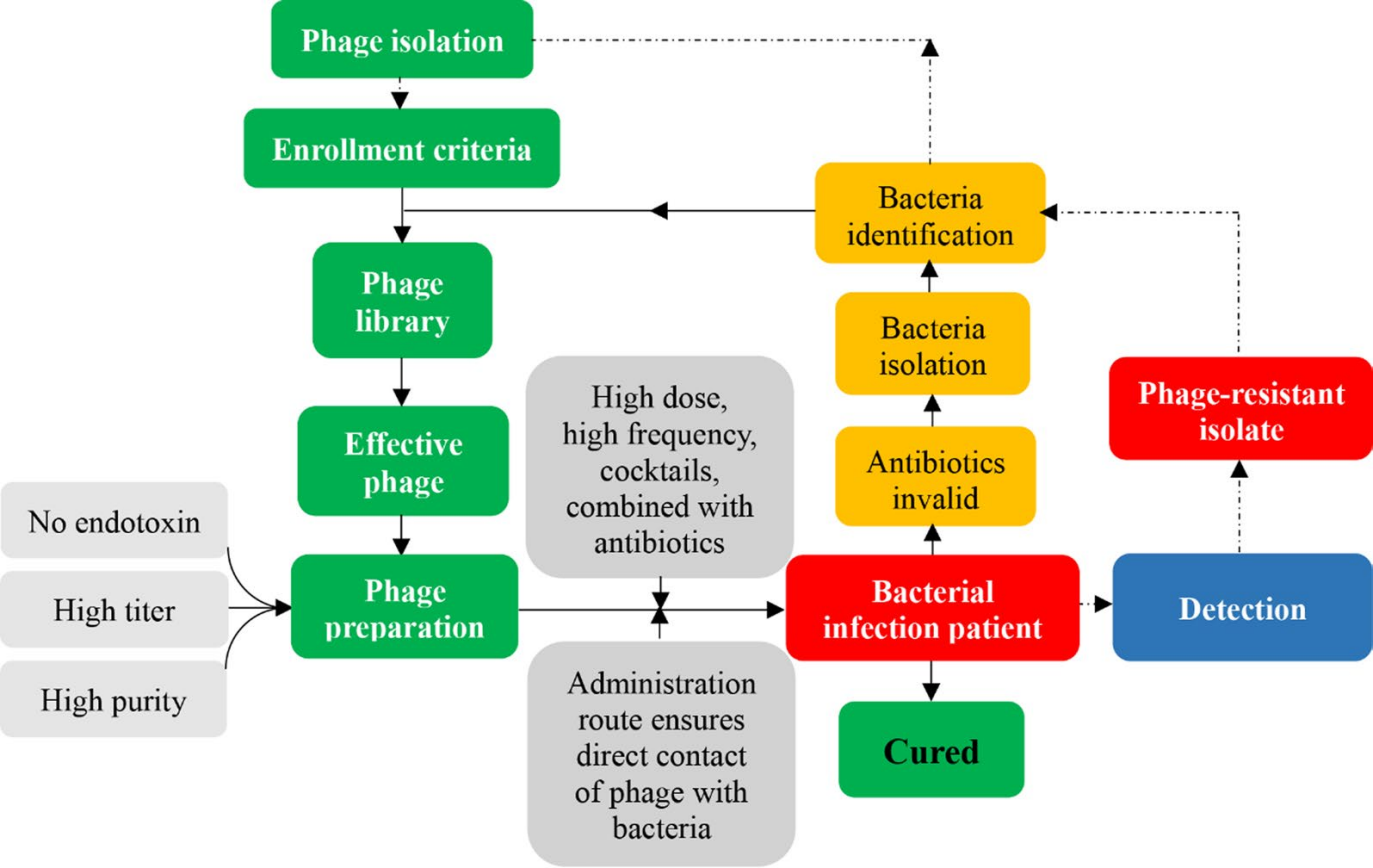
The Whole Streamlined Procedure of Phage Therapy in Clinical Practice. The criteria for enrolling phages for therapy should be established, and a library containing different phages should be established in advance. Antibiotic drugs should be proven ineffective for treatment of the bacterial infection, and the pathogen should be isolated and identified for further use in screening its corresponding effective phage in the library. Phage preparations that are free of endotoxin with a high titer and high purity should be generated. Administration strategies, including high dose, high frequency, cocktails, and combination with antibiotics, should be prioritized. The administration route should ensure direct contact of the phage with bacterium. The emergence of phage-resistant isolates and improvement of the infection should be monitored in a timely manner

In summary, as shown in Fig. 1, the construction of a phage library containing different phages that are effective against drug-resistant bacteria is the foundation for successful phage therapy. Phage inclusion criteria should be established. At the same time, for the treatment of bacterial infections, the pathogen should be cultured and then further identified in combination with other methods, including nonculture-based methods. Additionally, antibiotics should be proven to be ineffective. Based on these points, other important steps include identifying a corresponding effective phage to which the pathogen is sensitive, generating specific phage preparations, and choosing an administration strategy with the aim of mediating sufficient direct contact of the phage with the pathogen. Simultaneously, phage-resistant isolate detection and phage storage, transport, and administration routes were analyzed, with recommendations. The synergistic effect of phage therapy combined with antibiotics still needs to be explored to achieve the best clinical outcomes [59]. We hope that phage therapy will enter the clinic as soon as possible.

## Data Availability

The datasets used and/or analyzed during the current study are available from the corresponding author on reasonable request.
